# Mental workload, musculoskeletal disorders, and associated factors among international transport truck drivers

**DOI:** 10.47626/1679-4435-2023-1083

**Published:** 2024-09-24

**Authors:** Eduardo Timm Maciel, Vitoria Hamdan Padilha, Susane Graup, Alexandre Crespo Coelho da Silva Pinto

**Affiliations:** 1 Fisioterapia, Centro de Ciências da Saúde, Universidade Federal do Pampa (UNIPAMPA), Uruguaiana, RS, Brazil; 2 Educação Física, Centro de Ciências da Saúde, UNIPAMPA, Uruguaiana, RS, Brazil

**Keywords:** mental health, occupational health, workplace, transportation, saúde mental, saúde do trabalhador, local de trabalho, meios de transporte

## Abstract

**Introduction:**

Many health changes are related to how individuals respond to work events that are influenced by organizational, physical, and human factors.

**Objectives:**

We aim to evaluate the mental workload, the manifestation of musculoskeletal disorders, the associated factors, and their relationships among international transport truck drivers.

**Methods:**

This is a descriptive, cross-sectional diagnostic study of 70 truck drivers who entered the intermodal terminal of the city of Uruguaiana, state of Rio Grande do Sul, Brazil, in January 2021. Participants completed the NASA Task Load Index questionnaire and the Nordic Musculoskeletal Symptom Questionnaire, along with questions about work characteristics and health care.

**Results:**

The results showed that most truck drivers have a high mental workload (90.0%) and have experienced musculoskeletal symptoms in the past 12 months (72.9%). In addition, this occupational group has characteristics that are predictive of health problems, as most work over 10 hours a day (61.5%). The results also show a correlation between mental workload, hours worked, and lower back injuries (p < 0.05). There is also an association between hours worked and the occurrence of musculoskeletal disorders in the neck, shoulders, and lower back in the past 12 months and in the shoulders and lower back in the past 7 days, especially for those who work over 12 hours a day.

**Conclusions:**

The population studied had a high mental workload and a high prevalence of musculoskeletal disorders as well as occupational characteristics predictive of harm to both physical and mental health.

## Abstract (pt)

**Introdução:**

Muitas alterações de saúde estão associadas à forma como os indivíduos reagem aos eventos do trabalho, estando vinculadas a fatores organizacionais, físicos e humanos.

**Objetivos:**

Avaliar a carga mental de trabalho, a manifestação de distúrbios musculoesqueléticos, os fatores associados e suas relações em caminhoneiros de transportes internacionais.

**Métodos:**

Trata-se de um estudo descritivo diagnóstico de corte transversal realizado no Porto Seco Rodoviário do Município de Uruguaiana, estado do Rio Grande do Sul, durante o mês de janeiro de 2021, no qual foram avaliados 70 caminhoneiros. Os participantes responderam ao Questionário NASA Task Load Index e ao Questionário Nórdico de Sintomas Osteomusculares, além de questões acerca das características do trabalho e cuidados com a saúde.

**Resultados:**

Os resultados demonstraram que a maioria dos caminhoneiros apresentou elevada carga mental de trabalho (90%) e apresentou sintomatologias musculoesqueléticas nos últimos 12 meses (72,9%). Ademais, a categoria profissional apresenta características preditoras de prejuízos à saúde, uma vez que a maioria trabalha mais de 10 horas por dia (61,5%). Ainda, os resultados indicam correlação entre a carga mental de trabalho e as horas trabalhadas e as injúrias lombares (p < 0,05). Também houve correlação entre as horas trabalhadas e a manifestação de distúrbios musculoesqueléticos em pescoço, ombros e lombar nos últimos 12 meses e ombros e lombar nos últimos 7 dias para indivíduos com jornadas de trabalho diárias superiores a 12 horas.

**Conclusões:**

A população estudada apresentou elevada carga mental de trabalho e alta prevalência de distúrbios musculoesqueléticos, bem como características profissionais preditoras de prejuízos à saúde física e mental.

## INTRODUCTION

Mental workload (MWL) is a multidimensional construct defined as the interaction between the cognitive demands of a task, the characteristics of the person, and the characteristics of the situation.^1^ The basic concept of MWL is easily understood by analogy with muscular effort. Two examples can illustrate this concept: in the first, the individual exerts the maximum instantaneous force on a predetermined load; in the second, the amount of work done over a period is assessed, thereby determining the rates of muscular workload. In both cases, the load depends on the interaction between the demands of the rates and the capacity of the individual. Thus, performance is limited by the demands of the rate: either the operator meets the rates, or the rates exceed the capacity of the operator. MWL works similarly.^2^

Musculoskeletal disorders (MSDs) are conditions that affect workers in a variety of occupations.^3^ These disorders have a multifactorial etiology, influenced by socioeconomic and individual factors.^4^ They can cause inflammation and degeneration, primarily affecting structures such as muscles, nerves, tendons, joints, and cartilage, resulting in pain and functional limitations.^3^

Truck drivers are at high risk of developing these disorders because of excessive working hours and exposure to noise, vibration, high temperatures, forced postures and repetitive movements.^5^ In addition, factors such as poor road infrastructure and the high level of job responsibility, with few breaks for physical and mental recovery, can contribute to the onset of the diseases.^6^ This intense pace of work leads to greater physical, mental and emotional stress, which affects health, causes numerous disorders in the body, and consequently affects the quality of life.^7^

In this context, Ulhôa et al.^8^ conducted research in the southern and southeastern regions of Brazil, where more than half of the drivers (51.1%) reported low job satisfaction, which correlated with the development of minor psychological disorders such as depression, anxiety, fatigue, irritability, insomnia, and memory and concentration deficits. Similarly,

Ferreira & Alvarez^6^ showed that 43.89% of truck drivers reported that their current work activities affect their mental health, while 72.39% mentioned that their profession negatively affects their family and social relationships.

In turn, truck drivers continue to experience some of the highest rates of injury, lost workdays and compensation costs.^9^ Therefore, analyzing the work context of these workers is essential to understanding and preventing illness.^10^ Considering the data presented and the importance of the topic, this study aimed to analyze the MWL, the manifestation of MSDs and the associated factors among truck drivers at Porto Seco Rodoviário, in the city of Uruguaiana, state of Rio Grande do Sul.

## METHODS

This is a descriptive, diagnostic, cross-sectional study, in which Brazilian truck drivers accessing the Porto Seco Rodoviário in the city of Uruguaiana were evaluated. Uruguaiana has a strategic location because of the national dependence on imports and exports through the highways of the countries that make up the Southern Common Market (MERCOSUR). According to the Brazilian Association of International Transport (ABTI),^11^ between July 2019 and June 2020, 113,862 trucks crossed the border between Uruguaiana (Brazil) and Paso de los Libres (Argentina), with the main intention of exporting Brazilian production.

Data collection was conducted on-site over 3 days in January 2021, individually and by previously trained researchers. Considering the operational difficulties caused by the COVID-19 pandemic, measures were taken to prevent and manage research activities, to ensure basic health measures, to minimize damage and potential risks, and to ensure the integrity of the participants and the research team. In this context, researchers wore lab coats, PFF2 masks plus face shields, and sanitized their hands and materials with 70% alcohol before interviews. Respondents were also provided with disposable masks and hand sanitizer.

Ninety-seven truck drivers were invited to participate in the study, and 70 volunteered and met the inclusion criteria. To be included in the sample, the truck driver had to meet the following inclusion criteria: a) agree to voluntarily participate in the study; b) be at least 21 years old; c) have at least 2 years of professional experience; and d) be of Brazilian nationality. All ethical principles were adhered to in accordance with the Declaration of Helsinki (2008) and Resolution no. 466/12 of the Brazilian National Health Council. The research protocol was approved by the research ethics committee of the Universidade Federal do Pampa under opinion number no. 4.458.022.

The NASA Task Load Index (NASA-TLX) questionnaire was used to assess MWL. Developed by Hart & Staveland,^12^ NASA-TLX is a multidimensional rating scale that provides an overall MWL score based on a weighted average of ratings in six subscales: mental demand, physical demand, temporal demand, own performance (perceived performance), effort, and frustration.^2^ The sum of the questions allows to classify the individual as follows: from 0 to 20, low mental load; from 21 to 40, some mental load; from 41 to 60, moderate mental load; from 61 to 80, high mental load; and from 81 to 100, unbearable mental load. For bivariate analysis, the categories “low mental load” and “some mental load” were reclassified as “low mental load,” and the remaining categories were grouped into the “high mental load” category.

The Nordic Musculoskeletal Questionnaire (NMQ), validated for Brazil, was used to assess the incidence of MSDs. The NMQ was developed with the aim of standardizing the measurement of reports of musculoskeletal symptoms and thus facilitating the comparison of results between studies. The authors of this questionnaire do not recommend it as a basis for clinical diagnosis, but for the identification of MSDs. As such, it can be an important diagnostic tool for the work environment or workplace. There are three versions of the NMQ: a general version that covers all anatomical regions, and two specific versions for the lumbar region and the neck/shoulder region.^13^ The general version of the NMQ is presented in this study.

Work characteristics and health care were assessed by using an instrument developed specifically for this study to assess daily work hours and work experience, as well as daily use of medications, absenteeism because of MSDs, and use of specialized health care.

Data were first analyzed by using descriptive statistics, including frequency measures, mean (for quantitative variables), and standard deviation (SD). Tercile value was used to categorize age group, years of experience as a truck driver, and daily working hours. Pearson’s test was used to determine the correlation between numerical variables. Association between MWL and other study variables was tested using the chi-square test, with a significance level of 5% (p < 0.05).

## RESULTS

Sample consisted of 70 truck drivers with a mean age of 47.23 ± 11.24 years. Descriptive values of the variables analyzed are presented in Table 1. Therefore, truck drivers work an average of 11.9 hours a day, have 17.9 years of experience, and have a high MWL (64.9 points).

**Table 1:** Descriptive values of the analyzed variables of truck drivers at Porto Seco Rodoviário in the city of Uruguaiana. Uruguaiana, state of Rio Grande do Sul, 2021

Variables	Mean	SD
Age (years)	47.23	11.24
Length of time working (years)	17.89	11.26
Daily workload (hours)	11.93	3.27
MWL (in NASA-TLX points)	64.95	17.81

MWL = mental workload; SD = standard deviation; NASA-TLX = NASA Task Load Index.

Table 2 shows the frequency distribution of the variables and shows that all truck drivers are male (100%). The majority work over 10 hours a day (61.5%), have experienced MSDs in the last 12 months (72.9%) and have a high MWL (90%). In addition, 14.3% of the participants had to take time off work because of musculoskeletal symptoms.

**Table 2:** Frequency distribution of truck drivers according to the variables analyzed. Uruguaiana, state of Rio Grande do Sul, 2021

Variable	n	% (95%CI)
Age group (years)		
Up to 41.7	23	32.9 (21.9-43.9)
Between 41.8 and 53	26	37.1 (25.8-48.4)
Above 53	21	30.0 (19.3-40.7)
Length of time working as a truck driver (years)		
Up to 11	24	34.3 (23.2-45.4)
Between 11 and 22	25	35.7 (24.5-47.0)
Above 22	21	30.0 (19.3-40.7)
Daily workload (years)		
Up to 10	27	38.6 (27.2-50.0)
Between 10 and 12	23	32.9 (21.9-43.9)
Above 12	20	28.6 (18.0-39.2)
Mental workload		
Low	7	10.0 (2.9-17.0)
High	63	90.0 (83.1-97.0)
Pain/discomfort (last 7 days)		
Yes	33	47.1 (30.1-64.1)
No	37	52.9 (36.8-68.9)
Pain/discomfort (last 12 months)		
Yes	51	72.9 (60.7-85.1)
No	19	27.1 (71-47.1)
Have you taken time off work because of pain/discomfort (last 12 months)?		
Yes	10	14.3 (0.0-35.9)
No	60	85.7 (76.8-94.5)
Have you gone to a health professional because of pain/discomfort (last 12 months)?		
Yes	18	25.7 (5.5-45.9)
No	52	74.3 (62.4-86.2)
Do you take any medication on a daily basis?		
Yes	23	32.9 (13.7-52.1)
No	47	67.1 (53.6-80.5)

CI = confidence interval.

**Figure 1:**
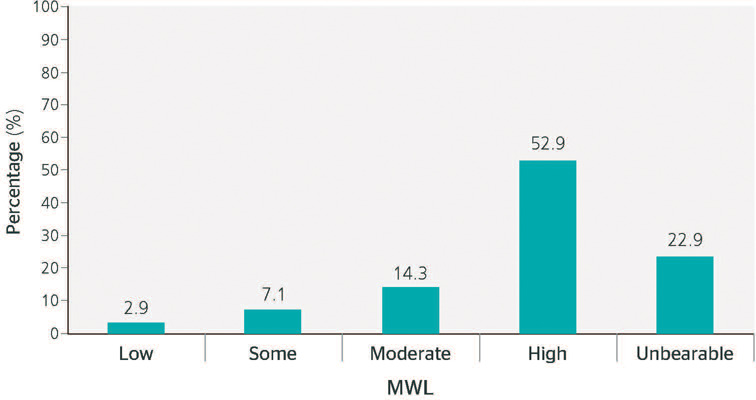
Frequency distribution of the mental workload (MWL) classification of the evaluated truck drivers. Uruguaiana, state of Rio Grande do Sul, 2021.

Figure 1 shows the classification of the MWL scores obtained from the truck drivers. Most respondents had a high level of MWL, with “high” and “unbearable” being the most common classifications.

**Figure 2:**
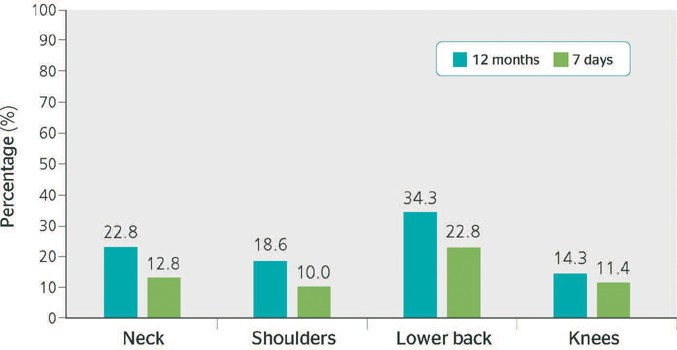
Body distribution of the prevalence of musculoskeletal disorders (MSDs) among the assessed truck drivers during the 12 months and 7 days preceding the assessment. Uruguaiana, state of Rio Grande do Sul, 2021.

Figure 2, which shows the distribution of MSDs by body region, indicates that the lumbar and neck regions had the highest prevalence of disorders in both periods analyzed.

When analyzing the correlation between the study variables and MWL, a significant correlation was found between MWL, and the number of hours worked a day (r = 0.432; p = 0.01). No correlations were found between MWL and any of the other study variables. When analyzing the association between categorical variables and categorized MWL, no significant associations were found, except for the presence of lower back pain in the last 7 days. In this regard, 56.2% of individuals reporting low back pain had an MWL categorized as intolerable (p = 0.002).

When considering the association between MSDs and the other study variables, only daily working hours were found to be significantly associated with neck, shoulder and lower back pain in the past 12 months and shoulder and lower back pain in the past 7 days (p < 0.05) (Table 3). In this regard, 50% of respondents with neck pain (p = 0.048), 61.5% of those with shoulder pain (p = 0.014), and 54.2% of those with lower back pain (p = 0.002) in the past 12 months worked over 12 hours a day. When analyzing the association with the frequency of pain in the last 7 days, 71.4% of those who reported shoulder pain (p = 0.030) and 56.2% of those who reported lower back pain (p = 0.018) also had the highest category of daily working hours (over 12 hours).

**Table 3:** Association between musculoskeletal disorders (MSDs) and daily working hours. Uruguaiana, state of Rio Grande do Sul, 2021

Variables	Daily workload (hours), n (%)			p value
	Up to 10	From 10 to 12	Over 12	
Neck pain over the last 12 months				
Yes	6 (37.5)	2 (12.5)	8 (50.0)	0.048*
No	21 (38.9)	21 (38.9)	12 (22.2)	
Shoulder pain over the last 12 months				
Yes	3 (23.1)	2 (15.4)	8 (61.5)	0.014*
No	24 (42.1)	21 (36.8)	12 (21.1)	
Lower back pain over the last 12 months				
Yes	7 (29.2)	4 (16.7)	13 (54.2)	0.002*
No	20 (43.5)	19 (41.3)	7 (15.2)	
Knee pain over the last 12 months				
Yes	4 (40.0)	3 (30.0)	3 (30.0)	0.978
No	23 (38.3)	20 (33.3)	17 (28.3)	
Neck pain over the last 7 days				
Yes	6 (66.7)	0 (0.0)	3 (33.3)	0.061
No	21 (34.4)	23 (37.7)	17 (27.9)	
Shoulder pain over the last 7 days				
Yes	1 (14.3)	1 (14.3)	5 (71.4)	0.030*
No	26 (41.3)	22 (34.9)	15 (23.8)	
Lower back pain over the last 7 days				
Yes	3 (18.8)	4 (25.0)	9 (56.2)	0.018*
No	24 (44.4)	19 (35.2)	11 (20.4)	
Knee pain over the last 7 days				
Yes	2 (25.0)	3 (37.5)	3 (37.5)	0.690
No	25 (40.3)	20 (32.3)	17 (27.4)	

*Significant value.

## DISCUSSION

In terms of sociodemographic variables (age, gender, time in the profession, and daily work hours), the results were similar to those obtained in a 2019 study by the Brazilian National Confederation of Transport (CNT),^14^ which characterized the profile of Brazilian truck drivers. The CNT results showed that 99.5% of Brazilian truck drivers are male, with an average age of 44.8 years, an average time in the profession of 18.8 years, and a daily work schedule of 11.5 hours.

Most individuals in this study had significant levels of MWL. In addition, a significant correlation was found between MWL, and the number of hours worked a day. This is because driving involves extreme fluctuations in MWL because of the considerable number of simultaneous factors that can affect the driver’s mind, such as interaction with in-vehicle devices and changes in driving demands, including distractions and traffic variations, which may result in insufficient time to process information.^15^

In addition, working conditions are characterized by long working hours, high pressure to meet deadlines, lack of rest breaks, irregular working hours, night shifts, unhealthy eating habits, and high consumption of alcohol and sleep-inhibiting drugs.^10^ Since anxiety, depression, substance abuse, and daily stress are among the most common causes of work-related mental illness, this is a concerning situation.^16^

We found 72.9% of respondents had experienced MSDs in at least one body part over the past 12 months, and 47.1% over the past 7 days. These data contrast with those obtained by Sekkay et al.^17^ when comparing risk factor exposure among 249 Canadian long-haul and short-haul truck drivers, where 43.1% of drivers reported discomfort in the past 12 months and only 26.8% in the past 7 days, with the highest prevalence found among long-haul drivers. In addition, Lemos et al.^18^ found a prevalence of 53.5% for musculoskeletal pain, most commonly in the lumbar spine.

Our results also reveal that the lumbar region is the most affected in both periods analyzed, with an incidence of 34.4% in the 12 months prior to the evaluation, along with a significant correlation with the highest MWL values. Cross-sectional studies conducted in different parts of the world have reported that 60% of truck drivers in the United Kingdom, 59% in São Paulo, Brazil, 73.5% in India, 88.7% in Tanzania, and 62.1% in Nagpur, India, reported lower back pain.^19^ Furthermore, in a similar population and using the NMQ, Nazerian et al.^20^ found a prevalence of lower back pain of 57.1% among 384 Turkish truck drivers, also over a 12-month period.

This high prevalence may be related to the fact that drivers sit in the same position for long periods of time, which increases the likelihood of lower back pain.^4^ However, this discomfort may be related not only to poor posture but also to truck maintenance activities such as lifting the toolbox, spare tire, and heavy tools.^20^ Psychosocial factors are also important causes of lower back pain,^21^ such as poor sleep, stress, tension or fatigue, and fear of being robbed, dying, getting sick, or having an accident on the job.^18^

In terms of physical effects, when looking at the association between MSDs and other study variables, daily working hours were found to be significantly associated with neck, shoulder, and lower back pain over the past 12 months and shoulder and lower back pain over the past 7 days. Similarly, Sa-Ngiamsak et al.^22^ found that long-haul truck drivers had a significantly higher prevalence of neck pain and more frequent lower back problems than short-haul drivers. In addition, Andrusaitis et al.^23^ found that for every hour a truck driver works each day, the risk of lower back pain increases by 7%. This suggests that the daily work load that truck drivers are exposed to leads to physiological fatigue that affects muscle function, joint range of motion, and adaptability.^24^

In addition, about one-third of the sample reported daily use of some type of medication. Girotto et al.^25^ and Belan et al.^26^ found incidences of 21.1% and 43.75%. These percentages are similar to those found in the present study, which also evaluated Brazilian truck drivers. In addition, Girotto et al.^25^ found in a sample of 665 truck drivers at the Porto de Paranaguá, located in the city of Paranaguá, state of Paraná, that certain occupational characteristics were associated with continuous medication use, such as longer driving experience, truck ownership, and lack of formal employment ties.

Despite the high prevalence of MSDs and ongoing medication use in this population, only 25.7% of respondents had seen a specialist in the previous 12 months. The incompatibility of work and duty hours and the perception of invulnerability among men contribute to truck drivers’ neglect of their health. In addition, the truck drivers studied by Masson and Monteiro^28^ also reported that they put their health “on the back burner,” with many participants stating that they had not visited a doctor in a long time, did not use health services while traveling, and did not have time to take care of their health. In addition, health initiatives for this population are rare and isolated.

The present study had some limitations, such as the number of participants. Due to the new social configurations brought about by the COVID-19 pandemic, our access to truck drivers was restricted. Considering that physical activity is a protective factor for physical and mental health, this could provide valuable information for a better understanding of the health status of the workers. Therefore, we suggest that the study should be replicated with a larger population and that other issues affecting the physical and mental well-being of truck drivers should be investigated.

## CONCLUSIONS

Based on the results, it can be concluded that the prevalence of high MWL is increased among the analyzed truck drivers, as well as the prevalence of MSDs, especially in lower back.

With this in mind, we recommend the development of regular interventions aimed at this professional niche, focusing on health promotion and prevention proposals, as well as the early diagnosis of existing problems. In this process, the specificities of this population must be considered in order to find new approaches that effectively integrate them into a healthier and more satisfying routine.
